# Comprehensive effects of interdecadal change of sea surface temperature increase in the Indo-Pacific Ocean on the warming-wetting of the Qinghai–Tibet Plateau

**DOI:** 10.1038/s41598-022-26465-8

**Published:** 2022-12-24

**Authors:** Na Dong, Xiangde Xu, Wenyue Cai, Tianliang Zhao, Chan Sun

**Affiliations:** 1 Department of Atmospheric and Oceanic Sciences, Institute of Atmospheric Sciences, Fudan University, Shanghai, China; 2 State Key Laboratory of Severe Weather, Chinese Academy of Meteorological Sciences, Beijing, 100081 China; 3 Nanjing University of Information Science and Technology, Nanjing, 210044 China; 4 National Satellite Meteorological Center, China Meteorological Administration, Beijing, 100081 China

**Keywords:** Climate sciences, Environmental sciences

## Abstract

The correlation characteristics between anomalous changes in summer precipitation on the Qinghai–Tibet Plateau (QTP) and the high-impact areas of global sea-surface temperature (SST) are mainly studied in this paper. The results show that the interdecadal change of the regional “warming-wetting” in China is especially prominent in the northern part of the main body of the QTP, which is therefore identified as the high-value area of precipitation variability. Investigations have revealed that the high-value areas of summer precipitation variability in the northern QTP are significantly correlated with four high-value areas of SST variability, namely the western North Pacific, the western Central Pacific, the Southwest Pacific, and the central Indian Ocean. In these four high-impact areas, a synchronous tendency is found in the SST increase and sea-surface specific humidity. Through the tracking analysis of the correlated vectors of the water vapor source for the warming-wetting of the QTP, it further confirms that the four high-value areas of SST variability in the Indo-Pacific Ocean are the major impact sources of water vapor transport for the warming-wetting of the QTP. Moreover, the comparison of the characteristics of various interdecadal global water vapor transport circulations show that from 1991 to 2020, the trans-equatorial water vapor transport from the Southern Hemisphere witnessed a notable increase, which furthermore suggests that the interdecadal change of SST increase in the Southwest Pacific and central Indian Ocean is the key reason for the warming-wetting of the QTP. In addition, a comprehensive image of high-impact marine water vapor sources for modulating the warming-wetting tendency in the QTP is proposed.

## Introduction

Under the background of global warming, a range of environmental issues are taking place in Asia, with those on the Qinghai–Tibet Plateau (QTP) especially prominent^1,2^. The QTP is one of the most sensitive regions to global warming and because of its special topography, the comprehensive effects of global warming has been amplified, thereby reinforcing the sea–land thermal contrast^3^. According to extensive observations and multiple key indices of current climate systems, the actual transpiration of the QTP has changed dramatically, exerting a considerable impact on the precipitation, lake water storage, and river runoff^4–6^ over the Plateau. The Intergovernmental Panel on Climate Change (IPCC) pointed out in its Sixth Assessment Report that based on the current observation statistics and numerical simulation results, extreme weather events such as high temperature and heavy precipitation on the QTP are showing an increasing tendency in the context of global warming. Frequent extreme events of high temperature are likely to be associated with human activities while the association between the increasing number of extreme events of heavy precipitation and human activities is unclear yet^7^.

The spatial distribution of precipitation in China is characterized by the gradual decrease from the southeast coast to the northwest inland areas. However, as proposed by quite a large number of studies, a climate transformation is taking place in Northwest China, namely from “warm-dry” to “warm-wet”^8–11^. Since 1987, a strong climate signal of transformation towards warm-wet has emerged in Xinjiang (mainly in the western part of Mt. Tianshan), where precipitation, glacial ablation and runoff have increased for consecutive years^12^ . Precipitation was observed to display an increasing tendency in 76% of the observation stations in Northwest China from 1997 to 2019. To be more specific, the growth rate of precipitation in the eastern part was higher than that in the western part of the Northwest^13^. Ma et al.^14^ have also spotted a transitional change towards higher precipitation in the eastern part of Northwest China since the beginning of the twenty-first century. On the QTP, continuous high precipitation has been observed, indicating a direction towards a warm-wet climate. According to statistics, from 1961 to 2021, the average decadal growth of the temperature increase rate was 0.36 °C/10 year and that of the annual precipitation was 10.2 mm/10 year^15,16^. Held and Soden^17^ proposed that the argument “wet gets wetter, dry gets dryer” does not agree with the case of the QTP because opposite tendencies appear in the northern and southern parts of the QTP, or more specifically, precipitation tends to increase prominently in the northern part and decrease in the southern part of the QTP^18^. Based on a great number of practical observation analyses and model estimation results, it is also found that both interdecadal changes and future climate tendencies indicate that with the development of global warming, precipitation on the QTP demonstrates an overall increasing tendency and the spatial distribution of the climate change has a nonuniform characteristic owing to the comprehensive land–air impact over the complicated megarelief^5^ .

As reported in earlier research, changes of arid and semiarid climates in Asia are related to the Pacific decadal oscillation (PDO). Jia and Zhang^19^ discovered that compared to 1960–1990, from 1990 to 2016, Northwest China, the QTP, the southeast of North China, and the majority of Northeast China went through obvious wetting: the west boundary line of extreme arid areas went eastward, both the south and north lines were shortened, and semiarid areas were transformed into subhumid areas gradually, all of which were related to the warm–cold phase change of the PDO. In addition, the warming-wetting of the QTP is mainly related to atmospheric dynamic and thermal structure changes, including sea-surface temperature (SST) increases, the northward movement of the Westerlies, the warming tendency of the middle and upper troposphere, and the occurrence frequency of low-level vortexes on the ground^19–23^. From the perspective of the hydrological cycle structure, the structural adjustment of water vapor transport of the surrounding oceans is also a key influencing factor for the warming-wetting of the QTP, since the QTP and the low-latitude marine monsoon activity area form a large triangular, fan-shaped water vapor impact area, and the North Indian Ocean, the Western Pacific, and the offshore areas constitute the QTP trans-hemisphere atmospheric water circulation system^24–26^, allowing the QTP to have four critical water vapor supply channels, namely the Indian channel, the South China Sea channel, the northern Bay of Bengal channel, and the Westerlies channel^27–29^. In addition, the summer SST anomalies in the Indian Ocean also have an impact on the precipitation of the QTP in the corresponding time; that is to say, when the Indian Ocean SSTs show a negative anomaly, the Indian summer monsoon becomes stronger, leading to heavier summer precipitation over the QTP^21,30,31^. In the summer following an El Niño event, an increase of the Indian Ocean SST can stimulate a Kelvin wave, resulting in a western Pacific anticyclone, which consequently impacts the low-level water vapor transport^32–34^.

The SST variation tendencies differ in different regions, and the range and mode of their impact vary as well, but the SST variations between different areas are somehow correlated^35^. The characteristics of anomalous SST variations are theoretically important to China’s long-term weather forecasts as well as climate predictions. As described in the abovementioned studies, the precipitation variation on the QTP has a significant correlation with the structural change characteristics of the marine impact sources. Hence, the following critical scientific questions are worthy of further discussion: how can we identify the ocean water vapor transport sources affecting the interdecadal variation of the warming-wetting of the QTP? How can we understand the comprehensive impacts of marine sensitive areas related to the interdecadal variation of the warming-wetting of the QTP? And how can we quantify the contribution of each ocean impact source to the warming-wetting of the QTP?

## Statistics and methods

### Statistics

To focus on the trend impact of the warming-wetting of the QTP from 1991 to 2020, the following statistics were used for analysis in this research: the 1° × 1° monthly average global SST statistics from 1991 to 2020 provided by the Japan Meteorological Agency^36^ (https://www.psl.noaa.gov/data/gridded/data.cobe.html), precipitation statistics from 1991 to 2020 (http://data.cma.cn/) obtained from 710 basic standard ground meteorological observation stations in the monthly value dataset of ground meteorological features in China (v3.0) provided by the National Meteorological Information Center of China Meteorological Administration, and the 1961–2020 monthly reanalysis data provided by the National Centers for Environmental Prediction (NCEP) and the National Center for Atmospheric Research (NCAR) (https://psl.noaa.gov/data/gridded/data.ncep.reanalysis.html), which include meteorological elements such as the two-meter specific humidity, ground pressure, zonal/meridional wind, specific humidity, air temperature, and vertical velocity, with a 2.5° × 2.5° horizontal resolution and 17 layers of vertical barometric surface pressures^37^.

## Methods

### Calculation methods of the whole-layer water vapor flux and related vectors

To discuss the modulating effects of significant areas of SST increase on the warming-wetting water vapor transport of the QTP and to analyse the structural characteristics of the water vapor transport channels related to SST anomalies in the Pacific and the Indian Ocean, vector methods relevant to water vapor transport were applied to track the effects of the water vapor sources and reveal the water vapor transport “paths” caused by SST anomalies in the significant areas of SST increase:

1 $$\mathrm{qu}=\frac{1}{\mathrm{g}}\underset{{\mathrm{p}}_{\mathrm{s}}}{\overset{\mathrm{p}}{\int }}\mathrm{qudp}$$

2 $$\mathrm{qv}=\frac{1}{\mathrm{g}}\underset{{\mathrm{p}}_{\mathrm{s}}}{\overset{\mathrm{p}}{\int }}\mathrm{qvdp}$$

where *g* represents gravitational acceleration, *u* and *v* represent zonal and meridional winds, respectively, *q* represents the specific humidity, *Ps* represents the ground pressure level, *P* represents the atmospheric top pressure, and *qu* and *qv* represent zonal and meridional water vapor fluxes, respectively^38^ . Next, the composite correlation vector is defined as:

3 $$\overrightarrow{R}(x,y)=\overrightarrow{i}{R}_{u}(x,y)+\overrightarrow{j}{R}_{v}(x,y)$$

where $\overrightarrow{R}$ denotes the composite correlation vector, ${R}_{u}$(*x, y*) denotes the correlation coefficient field of *qu* (i.e., the component of SST and zonal water vapor flux), and ${R}_{v}(x,y)$ denotes the correlation coefficient field of *qv* (i.e., the component of SST and meridional water vapor flux)^39^.

### Other methods

In addition to the above methods, calculation methods such as anomaly, linear trend estimate, correlation coefficients, variable standardization, and multiple regressions were also employed in this paper^39^. And the annual average value from 1991 to 2020 was taken as the climate average value, i.e. the normal value.

## Results

### Variation tendency and variability distribution of summer precipitation in China

**Figure 1:**
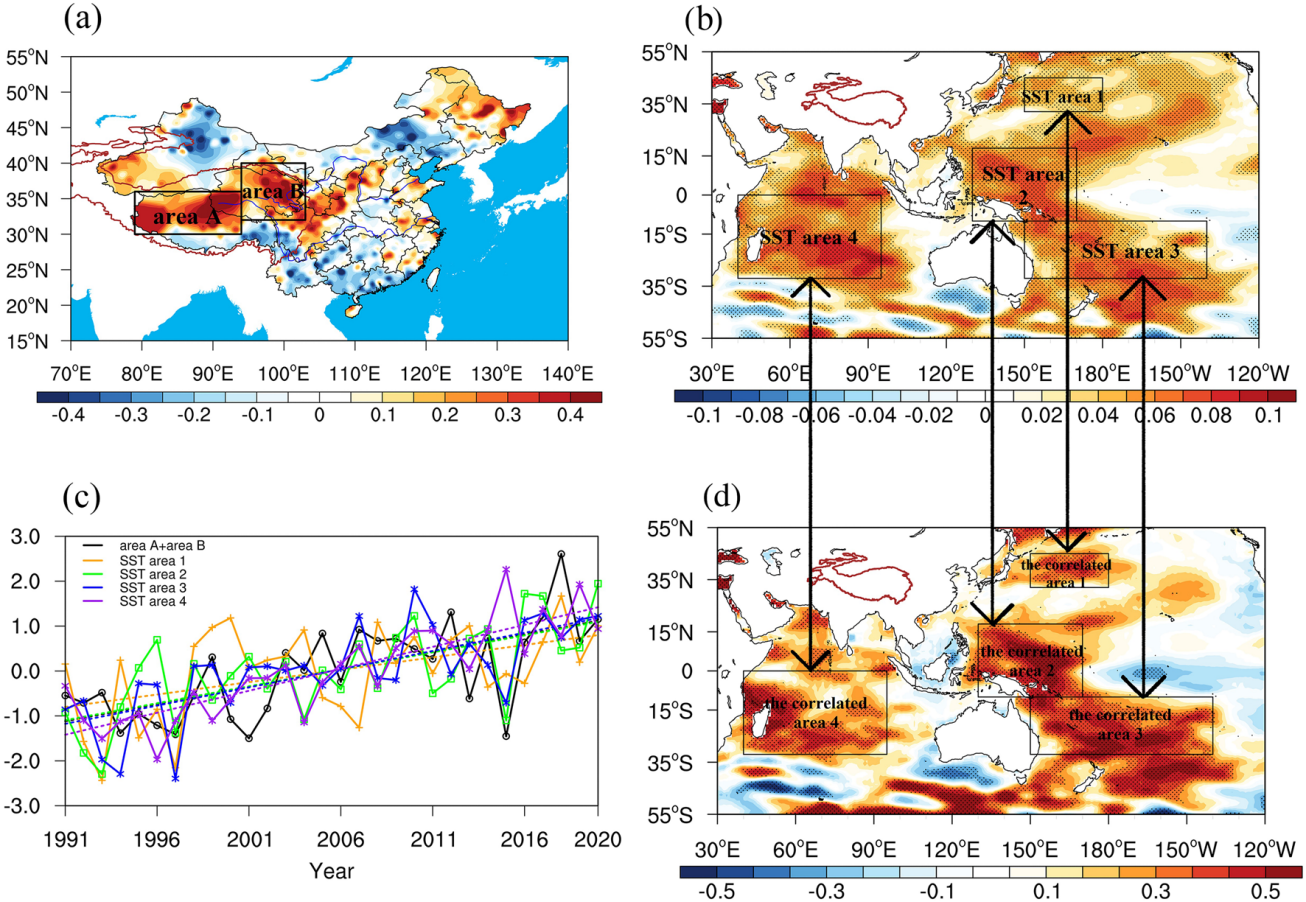
Summer of 1991–2020: (**a**) distribution characteristics of precipitation variability in mainland China (mm/10 year). (**b**) Distribution characteristics of global SST variability (°C/year). (**c**) Interannual variation curves of standardized precipitation in the high-value A and B areas of precipitation variability and standardized SST in high-value areas of SST variability (the correlation coefficients of precipitation and SST area 1, 2, 3, 4 are 0.40, 0.49, 0.61, and 0.49 respectively, dotted line: trend line of precipitation or SST). (**d**) Distribution correlation characteristics between the SST and precipitation in the high-value A and B areas of precipitation variability. (values over the 90% confidence level based on the student t-test are stippled and the color scale represents different values of the correlation coefficients.) The maps were generated with NCAR Command Language (NCL) Version 6.2.1 (http://www.ncl.ucar.edu/).

The characteristic of precipitation variability is an important index that reflects the regional response of the QTP to global climate change. In the context of global warming, summer precipitation over the QTP is affected by SST increases and west winds^31,40,41^, monsoons^42,43^, and structural changes in their circulations^44–47^. Meanwhile, the comprehensive land–air impact over the complicated megarelief results in a nonuniformity of the spatial distribution of climate change. The research results of our study reveal an opposite variation tendency of summertime precipitation variability in the south and north of the OTP from 1991 to 2020 (Fig. 1a). A decreasing tendency of precipitation was observed in the south and southeast of the QTP, while the central and the northeast parts of the QTP witnessed an increasing tendency. Hence the pattern of “dry south and wet north” is formed. This result is consistent with the analysis on the variation tendency of precipitation over the QTP by using the optimized variational TRMM precipitation products (Sun et al.^48^). Based on the distribution characteristics of high value areas of precipitation variability, the high-value areas of the QTP are divided into two areas, namely area A (northern Tibet) and area B (Qinghai and northern Sichuan). The composite analysis of precipitation in the two areas indicates that the maximum values of precipitation variability in both area A and area B occur in the summer (Table S1). From the interdecadal perspective (Fig. S3), the summer precipitation showed a reducing trend in area A from 1961 to 1991, while significant rising trend was identified from 1991 to 2020. As for area B, relatively weak decreasing trend was found from 1966 to 1991, while 1991–2020 also saw significant increasing trend. So after 1991, the summer precipitation in both area A and area B exhibited significant uplifting tendency. Notably, the overall increasing tendency of the summer precipitation variability in the two high-value areas of QTP (area A and area B) shows salient variation scopes, obviously higher than that for mainland China (Figure S2), suggesting that the warming-wetting phenomenon in China mainly occurs in the central and the northern part of the QTP, i.e., the high-value areas of precipitation variability (area A and area B).

### Correlation characteristics of summer precipitation in high variability areas of the QTP and SST changes in high-impact areas

On the temporal scale, the global average SST has increased in a nearly linear manner from the 1970s to the late twentieth century, consistent with the increase of greenhouse gases emitted by human activities, such as carbon dioxide. During this time, the 1980s and 1990s were two periods when the SST increased fastest^49^. Taking the Pacific and the Indian Ocean as key research objects, the 1991–2020 global SST variabilities were analysed (Fig. 1b) in this paper. It is found that four high-value areas of SST variability exist in the Pacific and the Indian Ocean, which are located in the western North Pacific (SST area 1), western Central Pacific (SST area 2), Southwest Pacific (SST area 3), and central Indian Ocean (SST area 4). Summer precipitation in the QTP’s key areas of warming-wetting shows a synchronous interannual increasing trend with the SSTs in the four high-value areas of SST variability. Their correlation coefficients reach 0.40, 0.49, 0.61, and 0.49 respectively (values over the 90% confidence level based on the student t-test) (Fig. 1c). Next, a correlation analysis was conducted on the summer precipitation of anomalous warm-wet key areas (area A and area B) on the QTP and the global SST in the corresponding period to identify the relevant high-value areas (Fig. 1d); the four key areas we identified agree with the spatial distribution of high-value areas of SST variability (Fig. 1b). Such analyses demonstrate a probable salient correlation between the significant areas of global SST increase and the warming-wetting phenomenon of the QTP, meaning the significant areas are likely to be the four SST high-impact areas for the warming-wetting on the QTP.

### Characteristics of the synchronous variation trends between SST increase and sea-surface water vapor in high-impact areas in summer

**Figure 2:**
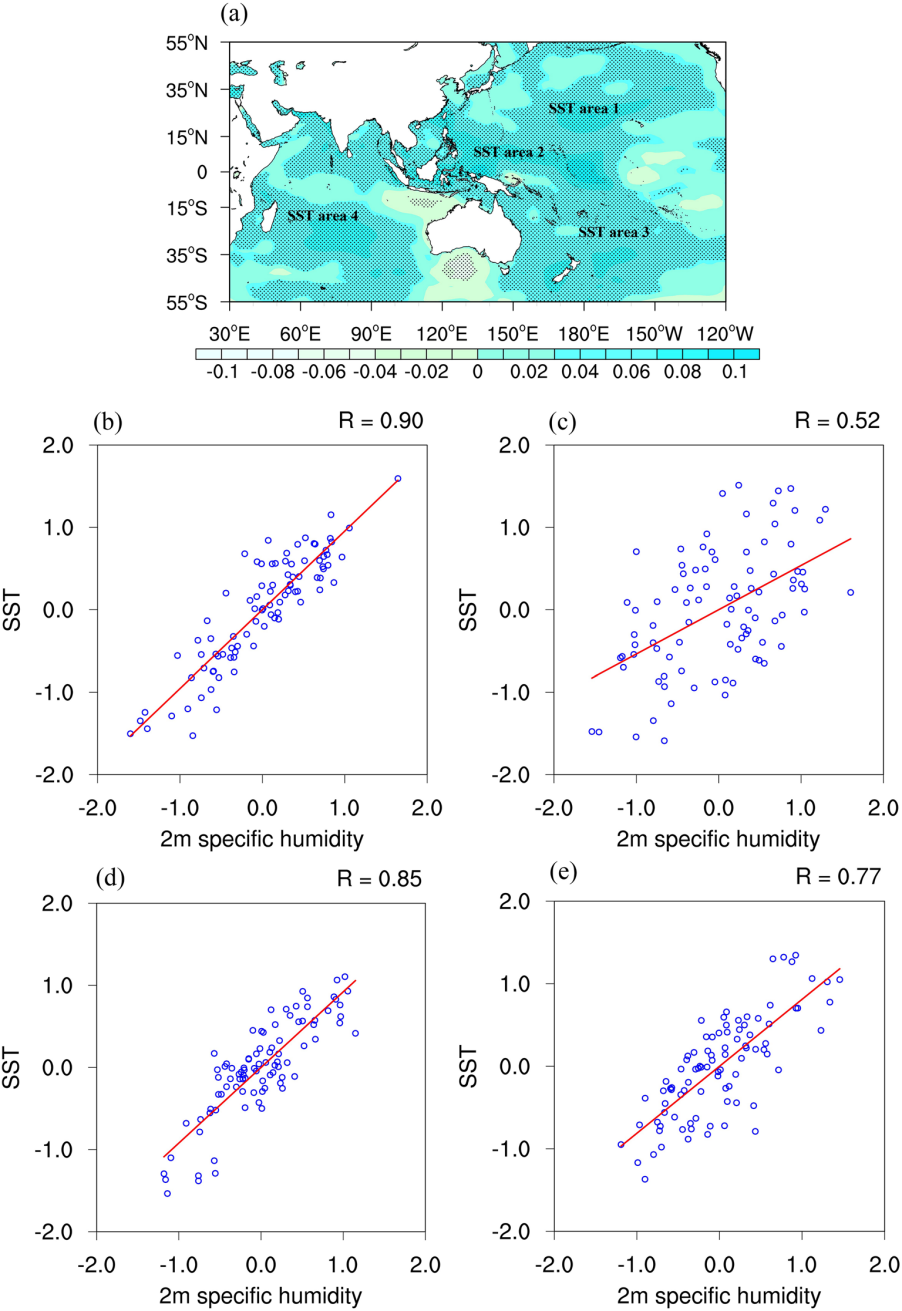
Summer of 1991–2020: (**a**) distribution characteristics of the global specific humidity variability at 2 m (g/kg/year; values over the 90% confidence level based on the student t-test are stippled). (**b–e**) Standardized scattered point distribution of specific humidity at 2 m and SST in the SST high-impact areas from June to August; (**b**) SST area 1, (**c**) SST area 2, (**d**) SST area 3, and (**e**) SST area 4. The maps were generated with NCAR Command Language (NCL) Version 6.2.1 (http://www.ncl.ucar.edu/).

The sea-surface temperature anomalies in the Pacific and the Indian Ocean have a significant impact on the water vapor budget of the QTP^50–53^. Multiscale modulations of monsoons and atmospheric circulations triggered by the sea–land thermal contrast in summer constitute the anomalous water vapor transport structure of the ocean water vapor sources, generating a climate teleconnection with the atmospheric water cycle over the QTP or the land. Therefore, SST increases in the high-impact areas of the Pacific and the Indian Ocean in summer might result in local water vapor anomalies, and inter-hemispheric teleconnection water vapor transport might be one of the vital factors that regulates precipitation on the QTP. In light of the above considerations, the global sea-surface specific humidity variability from 1991 to 2020 (Fig. 2a) was calculated. By comparing the four SST high-impact areas indicated in Fig. 1d, namely, the western North Pacific (the correlated area 1), the western Central Pacific (the correlated area 2), the Southwest Pacific (the correlated area 3), and the central Indian Ocean (the correlated area 4), it is found that all of them show high sea-surface specific humidity variability. Moreover, the sea-surface specific humidity variabilities in the four high-impact areas all demonstrate synchronous upward interannual trends (Fig. S4a–d). The correlation coefficients of the SST and the sea-surface specific humidity in the four high-impact areas are 0.90, 0.52, 0.85, and 0.77 respectively (values over the 90% confidence level based on the student t-test) (Fig. 2b–e). This result reveals that SST increases in the SST high-impact areas of the Pacific and the Indian Ocean in summer might lead to a tendency of anomalous high specific humidity in the local sea surface.

### How does the anomalous water vapor flow pattern caused by the SST increase in the high-impact areas affect the warming-wetting of the Plateau?

**Figure 3:**
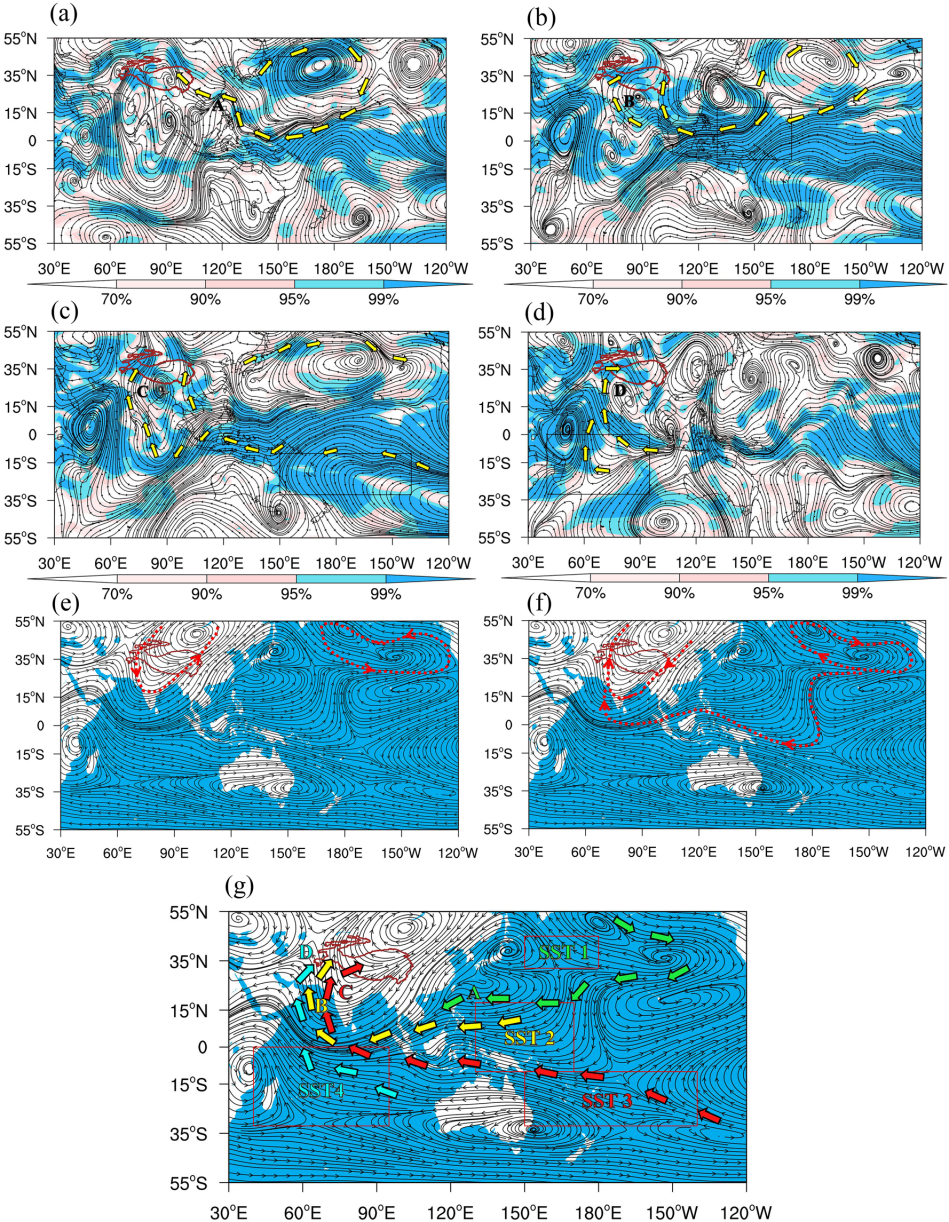
Distribution of correlation between standardized SST and surface-300 hPa water vapor flux in summer from 1991 to 2020. (**a**) SST area 1, (**b**) SST area 2, (**c**) SST area 3 and (**d**) SST area 4. Anomaly field of the whole-layer water vapor fluxes (**e**) for 1961–1990 and (**f**) for 1991–2020. (**g**) Comprehensive effect image of the water vapor transport structures in the four high-impact areas on the warming-wetting of QTP. The maps were generated with NCAR Command Language (NCL) Version 6.2.1 (http://www.ncl.ucar.edu/).

The QTP can capture anomalous warm and humid gas flows from the Indian Ocean, the South China Sea, the low-latitude western Pacific and other areas in the south through the “hollow heat island” effect and its continuous heat source^26^. The water vapor over the QTP has experienced dramatic changes under the effects of global warming, thus affecting the precipitation, lake water storage, and water vapor budget of the Plateau. According to the study by Zhang et al.^31^, increased water vapor in the west of the QTP in recent years has been the culprit for the precipitation growth in the central and western QTP. The stronger water vapor transport from the Indian Ocean to the Plateau boosts the water cycle on the QTP, which is the main process of water vapor for the “wetting” of the QTP. In this paper, the correlation vector method for water vapor transport is used as a tracer for the water vapor source to reveal any anomalous changes in the water vapor transport path due to SST anomalies in the high-impact areas. The aim here is to explore the regulating effect of the high-impact areas of SST increase on the water vapor transport in the cloud precipitation process on the QTP and further ascertain the structural characteristics of the water vapor transport channel correlated to the SST anomalies of the Pacific and Indian Ocean. According to Fig. 3a–d, the SST in area 1 and the whole-layer water vapor flux of East Asia over the same period demonstrate an anticyclonic circulation pattern, where a correlated water vapor flow A comes from the Pacific Ocean to the west and then turns northward to a high-value area of precipitation variability in North China and the northern QTP (Fig. 3a). The SST in area 2 and the whole-layer water vapor flux of East Asia over the same period demonstrate an anticyclonic circulation pattern in the Pacific, and the related water vapor flow B in the southwest is transported northward from the equatorial Pacific and turns westward to the high-value area of precipitation variability in the northern QTP (Fig. 3b). The SST in area 3 and the whole-layer water vapor flux of East Asia over the same period also demonstrate an anticyclonic circulation pattern in the Pacific Ocean, and the related water vapor flow C in the south crosses the equator, turns northward, and is transported to a high-value area of precipitation variability in the northern QTP (Fig. 3c). Again, the SST in area 4 and the water vapor flux of East Asia over the same period demonstrate an anticyclone circulation pattern in the central Indian Ocean, which is just on the southwest edge of the Pacific anticyclonic westward-extending circulation. After it crosses the equator, the related water vapor flow D is transported from the western QTP to a high-value area of precipitation variability in the northern Plateau (Fig. 3d).

### Relative contribution of the summertime SST increase in the high-impact areas to the warming-wetting of the QTP and an image of its comprehensive effect

Based on the previous discussions, the western North Pacific, the western Central Pacific, the Southwest Pacific and the central Indian Ocean are identified to be the key areas affecting the summer warming-wetting of the northern QTP in China. Under the combined effects of the SST high-impact areas, it is worth noting which areas have the most significant impact on the warming-wetting of the Plateau and what the contribution of each SST high-impact area to the warming-wetting is. Therefore, the relative contribution of the SST in these high-impact areas in summer to the warming-wetting of the QTP during the same period is further quantified in this section. A standardized multiple linear regression equation with the standardized SSTs in the four high-impact areas as the independent variables and the standardized precipitation in the warming-wetting sensitive areas of the QTP as the dependent variable is established as follows:

4 $$ {\text{y }} = \, 0.0{\text{85x}}_{{1}} + 0.0{\text{94x}}_{{2}} + 0.{\text{374x}}_{{3}} + 0.{\text{173x}}_{{4}} $$

In Eq. (4), *y* represents the standardized precipitation in the warming-wetting sensitive areas of the QTP, and *x*_*1*_*–x*_*4*_ denote the standardized SST (with *x*_*1*_ for area 1: the western North Pacific; *x*_*2*_ for area 2: the western central Pacific; *x*_*3*_ for area 3: the Southwest Pacific; and *x*_*4*_ for area 4: the central Indian Ocean).

The standardized regression coefficients can directly explain the share of contribution and configuration of the SST in the high-impact areas to the warming-wetting of the QTP under their combined effects. It can be learned from the relative contribution rates of the SST in the high-impact areas to the warming-wetting of the QTP that the Southwest Pacific is the most significant area for warming-wetting, with a relative contribution rate of 51%. The relative contribution of the central Indian Ocean is 24%. The impacts of the western North Pacific and the western central Pacific are secondary, with relative contribution rates of merely 12% and 13%, respectively. In summary, the trans-hemispheric SST increase, accompanied with energy and water vapor transport, can be determined to be a crucial and nonnegligible factor modulating the warming-wetting tendency of the QTP.

According to the Fifth Assessment Report by the IPCC, the global oceans are experiencing remarkable warming, with the fastest SST increase in the near-surface layer yet recorded^54^. Although global ocean warming is largely certain, there are great regional differences in the rate and magnitude of SST increase in time and space^55^. By comparing the anomaly field differences of the whole-layer water vapor fluxes from 1961 to 1990 and from 1991 to 2020, the differences in the trans-hemispheric ocean energy and water vapor transport circulation on interdecadal temporal scales under the influence of global warming are discussed in this paper to identify the large-scale circulation background for the formation of the warming-wetting of the QTP. It can be observed from the comparison between Fig. 3e,f that the circulation of the water vapor transport on the QTP affected by the anomaly field of the whole-layer water vapor fluxes from 1991 to 2020 was opposite to that from 1961 to 1990. That is, from 1961 to 1990, the water vapor transport fluxes from the Indian Ocean to the QTP and its East Asian region was shown as a north–south axial cyclonic circulation, with the southerly water vapor flow on its eastern side transported to the southern Plateau and the eastern region of China. In contrast, from 1991 to 2020, the water vapor transport fluxes of the Indian Ocean to the QTP and the East Asian region was exhibited as a north–south axial anticyclonic circulation. In this way, the southerly water vapor flow on its western side was transported to the western and central Plateau, while the northerly water vapor flow on its eastern side transported the Northwest Pacific water vapor flow from the east to the northern Plateau. Moreover, from 1961 to 1990, the anomaly field of the water vapor transport fluxes in the western Pacific was opposite to that from 1991 to 2020. Specifically, from 1991 to 2020, the anticyclonic circulation extended to the southern hemisphere, the trans-equatorial easterly water vapor flow on its southern edge passed through the Indian Ocean to the easterly flow of the southern edge of the anticyclonic circulation of the Plateau, and then was transported to the western and central part of the Plateau.

It reveals that the trans-equatorial water vapor transport in the Southern Hemisphere was significantly enhanced from 1991 to 2020, further indicating that the SST increase in the Southwest Pacific and the central Indian Ocean is the key reason for the warming-wetting of the QTP. In addition, the characteristics of the 500 hPa circulation situation were observed to be similar to those of the anomaly field of the whole-layer water vapor transport fluxes (Fig. S5a,b). The anticyclonic water vapor transport circulation in the Pacific and the Indian Ocean from 1991 to 2020 was favorable for the transport of warm-wet ocean water vapor to the western and central Plateau. Thereby, an image of the comprehensive effect of the water vapor transport structures in the four high-impact areas on the warming-wetting of the QTP is proposed, with the anomaly field of water vapor transport from 1991 to 2020 as the background (Fig. 3g).

## Discussion

To focus on the trend impact of the “warming-wetting” of the QTP from 1991 to 2020, the correlation characteristics between the anomalous summer precipitation variation on the QTP and global SST high-impact areas were studied. The correlation mechanisms of the climate anomalous warming-wetting of the QTP and the changes in ocean water vapor sources of the Indian Ocean and Pacific were researched from the perspective of atmospheric water circulation so as to verify that the areas of the Pacific and the Indian Ocean linked to the Asian continent might be the impact sources of water vapor transport for the warming-wetting of the QTP. The reasons for the reinforced warming-wetting of the QTP in summer were analysed and the following conclusions were reached:

1. From 1991 to 2020, the regional characteristics of warming-wetting of the QTP varied significantly in summer. The precipitation variabilities in the southern and northern Plateau showed opposite trends. Specifically, precipitation in the south and southeast of the QTP showed a decreasing trend, while the precipitation in the central and the northeast of the QTP exhibited an increasing trend. Hence the pattern of “dry south and wet north” is formed on the QTP.
2. There were four high-value areas of SST variability in the Pacific and the Indian Ocean during the summers from 1991 to 2020, namely, the western North Pacific, the western Central Pacific, the Southwest Pacific, and the central Indian Ocean. Through the correlation analysis of the summer precipitation over the QTP’s significant areas of warming and wetting (area A and area B) and the global SST during the same period, it is found that there are also four high-correlation areas in the Pacific Ocean and the Indian Ocean. And these four areas greatly coincide with the spatial distribution of the above four high-value areas of SST variability.
3. In summer, the spatial distribution characteristics of the high-value areas of SST variability are similar to those of the sea-surface specific humidity variability in the Pacific and the Indian Ocean. The SST increase and the sea-surface specific humidity in the four high-impact areas are featured by synchronous change trends. Their correlation coefficients reach 0.90, 0.52, 0.85 and 0.77, respectively (values over the 90% confidence level based on the student t-test). It is concluded that the SST increase in the high-impact areas of the Pacific and the Indian Ocean in summer can lead to enhanced regional sea-surface transpiration, which in turn results in the anomalous high humidity change trends of local sea-surface water vapor.
4. By analysing the correlated synthetic vectors between the SSTs in the four high-impact areas in the Pacific and the Indian Ocean and the whole-layer water vapor fluxes in East Asia during the same period in summer, it can be drawn that area 1 demonstrates an anticyclonic circulation pattern, where a correlated water vapor flow A, originally in the south flank of the area, comes from the Pacific Ocean to the west and then is transported from the southeast to the central and the northern QTP; area 2 is identified with an anticyclonic circulation pattern in the west of the central Pacific, and the related water vapor flow B comes from the equatorial Pacific, turns westward and then is transported from the southeast and the west to the central and the northern QTP; area 3 also demonstrates an anticyclonic circulation pattern in the southwest Pacific Ocean, and the related water vapor flow C crosses the equator, turns northward, and is transported from the southwest and the west to the central and northern QTP; area 4 is in the central Indian Ocean and just on the southwest edge of the Pacific anticyclonic westward-extending circulation. After it crosses the equator, the related water vapor flow D is transported from the western QTP to the central or northern QTP.
5. In summer, the contributions of the four high-impact areas of the SST increase to the warming-wetting of the QTP are significantly different. The contribution of each ocean impact source to the warming-wetting of the Plateau was identified as 51%, 24%, 12%, and 13%, respectively, by a standardized multiple linear regression analysis. Among them, the Southwest Pacific is the most significant area, suggesting that trans-hemispheric SST increases accompanied by energy and water vapor transport is a nonnegligible factor modulating the warming-wetting of the QTP. From 1991 to 2020, the trans-equatorial water vapor transport in the Southern Hemisphere was found to be significantly enhanced by comparing the characteristics of different interdecadal global water vapor transport circulation patterns. It illustrates that the SST increases in the Southwest Pacific and the central Indian Ocean are the primary causes for the warming-wetting of the QTP. The warming-wetting trend of the QTP can be modulated by trans-hemispheric water vapor transport and energy exchange in the above four high-impact areas with significant SST increases. Therefore, an image of the comprehensive effect of the high-impact oceanic water vapor sources modulating the warming-wetting trend of the QTP is proposed in this paper from the perspective of the interdecadal variability of summer monsoon circulation.
6. Does the phenomenon of warming and wetting over the QTP only exist in summer? By calculating the precipitation variation tendencies over the QTP in four seasons of spring, summer, autumn and winter from 1991 to 2020 (Figures S1), it is found that the spring precipitation over the QTP shows an increasing tendency, while in summer, opposite trends are observed between the south and the north QTP. As for autumn, an increasing trend is captured in the northeast and the north edge of the QTP, while the precipitation trend in the central and the south edge is the opposite. In winter, most part of the QTP exhibits a reducing trend in precipitation, but an upward trend is found both in the south edge and in the north edge of the QTP. The calculation of precipitation variabilities in area A and area B in four seasons shows that (Table S1) area A (central QTP) is accompanied with positive precipitation variabilities in spring and summer but negative values appear in autumn and winter. In area B (northeastern QTP), the precipitation variabilities are positive in spring, summer and autumn, and weak positive variability is observed in winter. Notably, although the warming and wetting of the QTP signals strong regional characteristics in different seasons, the precipitation variation trend still displays the pattern of “dry south and wet north” from the perspective of the overall variation trend in four seasons in mainland China. This is likely affected by the high impact areas of SST that are related to the warming and wetting of the QTP. The seasonal variations of the water vapor circulation structures formed by the above high impact areas of SST may be correlated, to some extent, with the tendency of “dry south and wet north” in mainland China. The conclusion needs to be further studied in the future.

## Supplementary Information

Supplementary Information.

## Data Availability

All datasets used in this study are publicly available. The Sea Surface Temperature data are retrieved from https://www.psl.noaa.gov/data/gridded/data.cobe.html. The precipitation statistics obtained by 710 basic standard ground meteorological observation stations in the monthly value dataset of ground meteorological features in China (v3.0) provided by the National Meteorological Information Center of China Meteorological Administration (http://data.cma.cn/). Reanalysis data provided by the National Centers for Environmental Prediction (NCEP) and the National Center for Atmospheric Research (NCAR) (https://psl.noaa.gov/data/gridded/data.ncep.reanalysis.html).
