# Supplementary material for: Comprehensive effects of interdecadal change of sea surface temperature increase in the Indo-Pacific Ocean on the warming-wetting of the Qinghai–Tibet Plateau
Source: Sci Rep. 2022 Dec 24;12:22306. doi: 10.1038/s41598-022-26465-8 (PMC9789985; doi:10.1038/s41598-022-26465-8)
Supplement: Supplementary file 1 — Supplementary Information. [file 41598_2022_26465_MOESM1_ESM.docx]

**Supplementary information**

**Comprehensive Effects of Interdecadal Change of Sea Surface Temperature Increase in the Indo-Pacific Ocean on the Warming-Wetting of the Qinghai–Tibet Plateau**

**Na Dong ^1,2^, Xiangde Xu ^2,*^ , Wenyue Cai ^2,*^, Tianliang Zhao ^3^&Chan Sun ^4^**

^1^ Department of Atmospheric and Oceanic Sciences & Institute of Atmospheric Sciences, Fudan University, Shanghai, China

^2^ State Key Laboratory of Severe Weather, Chinese Academy of Meteorological Sciences, Beijing 100081, China

^3^ Nanjing University of Information Science & Technology, Nanjing 210044, China

^4^ National Satellite Meteorological Center, China Meteorological Administration, Beijing 100081, China

**Other methods**

1. the vertically integrated moisture flux anomaly

In order to illustrate the impact of global warming, the differences in the trans-hemispheric ocean energy and water vapor transport circulation on interdecadal temporal scales, in this paper, the vertically integrated moisture flux anomaly from 1961 to 1990 and from 1991 to 2020 are calculated

equation: $\text{x}_{\text{dt}}=x_{t}-\overline{x},$ $\text{t}=1,2,\cdots,n$ (1)

meaning: reflect the situation of data deviation from the average value.

1. linear trend estimate:

Summer precipitation in China is affected by the comprehensive land–air impact over the complicated megarelief, and the spatial distribution of climate change is non-uniform, therefore, the linear trend of summer precipitation in mainland China was calculated by using the monthly mean precipitation data of 710 stations in China during 1991-2020 and formula (2). On the temporal scale, the global average SST has increased in a nearly linear manner from the 1970s to the late 20th century. The 1980s and 1990s were two periods when the SST increased fastest, therefore, this paper calculates the linear trend of global summer SST from 1991 to 2020 with the same method. The SST increases in the high-impact areas of the Pacific and the Indian Ocean in summer might result in local water vapor anomalies, and inter-hemispheric teleconnection water vapor transport might be one of the vital factors that regulates precipitation on the QTP. In light of the above considerations, this paper further calculates the trend of global sea-surface specific humidity in summer from 1991 to 2020.

If x_i_ is used to represent a climate variable with a sample size of n, and t_i_ is used to represent the time corresponding to x_i_, the linear regression equation between x_i_ and t_i_ is established:

$\hat{x}_{i}=a+bt_{i},\text{（}i=1,2,...,n)$ (2)

Equation (2) can be regarded as a special and simple form of linear regression. Its implication is to express the relationship between x and time t with a reasonable straight line. In equation (2), a is the regression constant and b is the regression coefficient. a and b can be estimated using the least square method. For the observation data x_i_ and the corresponding time t_i_, the least square estimation of regression coefficient b and constant a is

$\begin{matrix} \text{b}=\frac{\sum_{i=1}^{n} x_{i}t_{i}-\frac{1}{n}\left(\sum_{i=1}^{n} x_{i}\right)\left(\sum_{i=1}^{n} t_{i}\right)}{\sum_{i=1}^{n} t_{i}^{2}-\frac{1}{n}\left(\sum_{i=1}^{n} t_{i}\right)^{2}}, \\ \\ a=\overline{x}-b\overline{t}, \end{matrix}$ (3)

$$\overline{\text{x}}=\frac{1}{n}\sum_{i=1}^{n} x_{i},$$

$$\overline{t}=\frac{1}{n}\sum_{i=1}^{n} t_{i}$$

The regression coefficient b can represent the tendency of climate variable x. When the sign of b is positive, it means that x increases with time t; Conversely, when the sign of b is negative, it means that x decreases with time t. At the same time, the magnitude of b reflects the rate at which the variable x rises or falls.

1. correlation coefficients：

The purpose of this paper is to show that ① the summer precipitation in the QTP’s key areas of warming-wetting and the SSTs in the four high-value areas of SST variability are significantly correlated, and ② the SST increases in the high-impact areas of the Pacific and the Indian Ocean in summer might lead to a tendency of anomalous high specific humidity in the local sea surface. The correlation coefficient calculation method is adopted in this paper.

Pearson correlation coefficient is a statistic that describes the linear correlation between two random variables. It is generally referred to as correlation coefficient or point correlation coefficient for short and expressed by r.

There are two variables

x_1_,x_2_,...,x_n_ and y_1_,y_2_,...,y_n_,

The calculation formula of correlation coefficient is

$\text{r}=\frac{\sum_{i=1}^{n} (x_{i}-\overline{x})(y_{i}-\overline{y})}{\sqrt{\sum_{i=1}^{n} (x_{i}-\overline{x})^{2}}\sqrt{\sum_{i=1}^{n} (y_{i}-\overline{y})^{2}}}$ (4)

It is easy to prove that the value of correlation coefficient r ranges from -1.0 to +1.0, and the closer its absolute value is to 1, the closer the relationship between variables is. When r>0, it indicates that the two variables are positively correlated, and the closer to 1.0, the more significant the positive correlation is. When r<0, it indicates that the two variables are negatively correlated, and the closer they are to -1.0, the more significant the negative correlation is. When r=0, the two variables are independent of each other.

If the correlation analysis is for two columns of sample data, it is expressed as the relationship between different samples. If each column is a field, it is the spatial correlation, usually called the spatial correlation coefficient, also known as the similarity coefficient.

Of course, whether the calculated correlation coefficient is significant needs to be tested. The correlation coefficient r needs to pass the T-test with n-2 degrees of freedom, where n is the sample size:

$\text{t}=r\cdot\sqrt{(n-2)(1-r^{2})}$ (5)

1. variable standardization

Objective: Each meteorological element unit is different, and the mean value and standard deviation are different. To compare them at the same level, the standardization method is used to make them become unitless variables at the same level ---- standardized variables.

$\text{x}_{zt}=\frac{x_{t}-\overline{x}}{s_{x}},$ $\text{t}=1,2,...,n$ (6)

$s_{x}=\sqrt{\frac{1}{\text{n}}\sum_{t=1}^{n} \left(x_{t}-\overline{x}\right)^{2}},$ $\text{t}=1,2,\cdots n$ (7)

$s_{x}$ is mean square deviation, which describes the average situation of the difference between the data and the mean value in the sample and reflects the average variation degree of variables around the mean value.

1. multiple linear regression

Objective: In meteorological statistical forecasting, it is difficult to find a single factor with a good linear relationship with the forecast. In fact, the change of a meteorological element is related to multiple factors, so most of the regression analysis in meteorological statistical forecasting is carried out by multiple linear regression method.

Under the combined effects of the SST high-impact areas, it is worth noting which areas have the most significant impact on the warming-wetting of the Plateau and what the contribution of each SST high-impact area to the warming-wetting is.

Therefore, this paper quantifies the relative contribution of summer SSTs in high-impact areas to the warming-wetting of the QTP through standardized multiple linear regression method. With the standardized SST in the four high-impact areas as the independent variable and the standardized precipitation in the warming-wetting sensitive areas of the QTP as the dependent variable, a standardized multiple linear regression equation was established and then the standardized regression coefficients can directly explain the share of contribution of the SSTs in the high-impact areas to the warming-wetting of the QTP under their combined effects.

Basic concept: Multiple regression is to study the relationship between a predictor and multiple predictor factors. This paper mainly discusses the simple multiple linear regression. The analysis principle is exactly the same as that of unary linear regression analysis.

Regression model: It is assumed that the relationship between the prediction quantity y and p prediction factors is linear. In order to study the relationship between them, the following structural expression can be obtained by sampling n times:

$\begin{matrix} y_{1}=\beta_{0}+\beta_{1}x_{11}+\beta_{2}x_{12}+\cdot\cdot\cdot+\beta_{p}x_{1p}+e_{1} \\ y_{2}=\beta_{0}+\beta_{1}x_{21}+\beta_{2}x_{22}+\cdot\cdot\cdot+\beta_{p}x_{2p}+e_{2} \\ \cdot\cdot\cdot\cdot\cdot\cdot\cdot\cdot\cdot\cdot\cdot\cdot\cdot\cdot\cdot\cdot\cdot\cdot\cdot\cdot\cdot\cdot\cdot\cdot\cdot\cdot\cdot\cdot\cdot\cdot\cdot\cdot\cdot\cdot\cdot\cdot\cdot\cdot\cdot\cdot\cdot\cdot\cdot\cdot\cdot\cdot\cdot\cdot\\ y_{n}=\beta_{0}+\beta_{1}x_{n1}+\beta_{2}x_{n2}+\cdot\cdot\cdot+\beta_{p}x_{np}+e_{n} \end{matrix}$ (8)

Where $\beta_{\text{i}}$ is $p+1$ parameters to be estimated, $\text{x}_{i}$ is $\text{p}$ general variables, and $e_{i}$ is random errors ( independent variables ) that obeys the normal distribution. The above model can also be written as:

$\text{y}=X\beta+e$ (9)

$\text{y}=\left[\begin{matrix} y_{1} \\ y_{2} \\ \vdots\\ y_{n} \end{matrix}\right]$ , $\beta=\left[\begin{matrix} \beta_{0} \\ \beta_{1} \\ \vdots\\ \beta_{\text{p}} \end{matrix}\right]$ , $\text{e}=\left[\begin{matrix} e_{0} \\ e_{1} \\ \vdots\\ e_{\text{p}} \end{matrix}\right]$ (10)

$X$ is a factor matrix

$X=\left[\begin{matrix} 1 & \text{x}_{11} & \cdots& x_{1p} \\ 1 & x_{21} & \cdots& \text{x}_{2p} \\ \cdots& \cdots& \cdots& \cdots\\ 1 & x_{n1} & \cdots& x_{np} \end{matrix}\right]$ (11)

We obtained a set of samples of measured p variables, which were used to estimate the above regression model. The estimated equation obtained was the multiple linear regression equation, denoted as:

$\hat{\text{y}}=b_{0}+b_{1}x_{1}+b_{2}x_{2}+\cdots+b_{\text{p}}x_{p}$ (12)

Where $b_{i}$ is an estimate of $\beta_{i}$.

If the variables are standardized, the standardized linear regression equation can be obtained:

$\hat{\text{y}}_{\text{z}}=b_{\text{z1}}x_{z1}+b_{\text{z2}}x_{z2}+\cdots+b_{\text{zp}}x_{zp}$ (13)

After standardization, the contribution rate of each variable factor to the forecast quantity can be obtained by dividing the coefficient of each variable by the sum of the total coefficients.

Significance test of regression equation:

Assuming that there is no linear relationship between predictor and predictand, the regression coefficient should be 0.

Test the hypothesis：

$$H_{0}:\beta_{1}=\beta_{2}=\cdots=\beta_{P}=0$$

Calculating statistics

$F=\frac{\frac{U}{\text{p}}}{\frac{Q}{\text{n}-p-1}}$ (14)

According to the $F$ distribution with molecular degree of freedom $\text{p}$ and denominator degree of freedom $\text{n}-p-1$, at the significance level $\partial$, If $F>F_{\partial}$, the regression equation is significant.

Figure S1


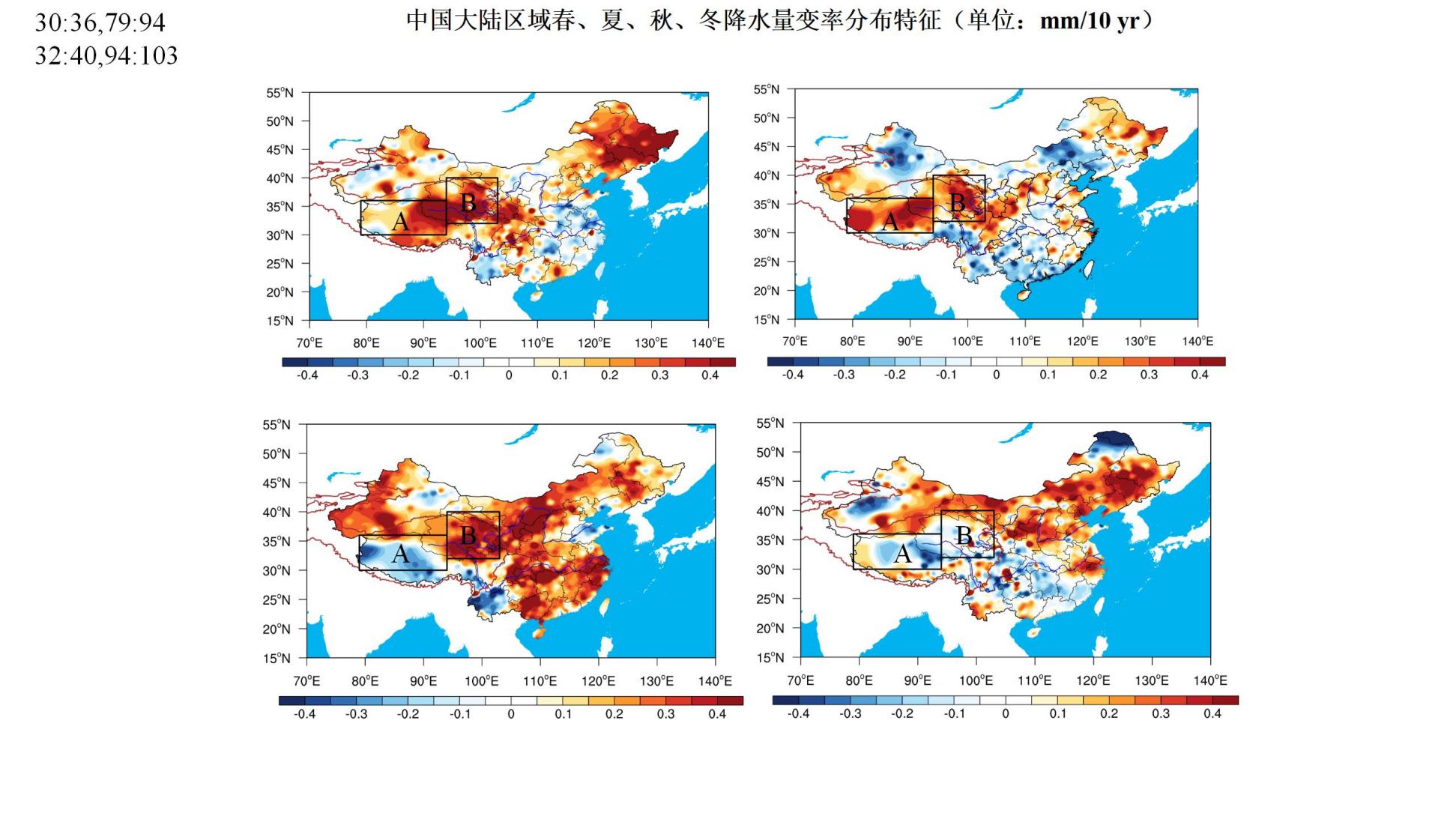


Figure S1: The distribution characteristics of precipitation variabilities in spring, summer, autumn and winter in mainland China from 1991 to 2020 (mm/10 yr). The maps were generated with NCAR Command Language (NCL) Version 6.2.1 (http://www.ncl.ucar.edu/).

Table S1


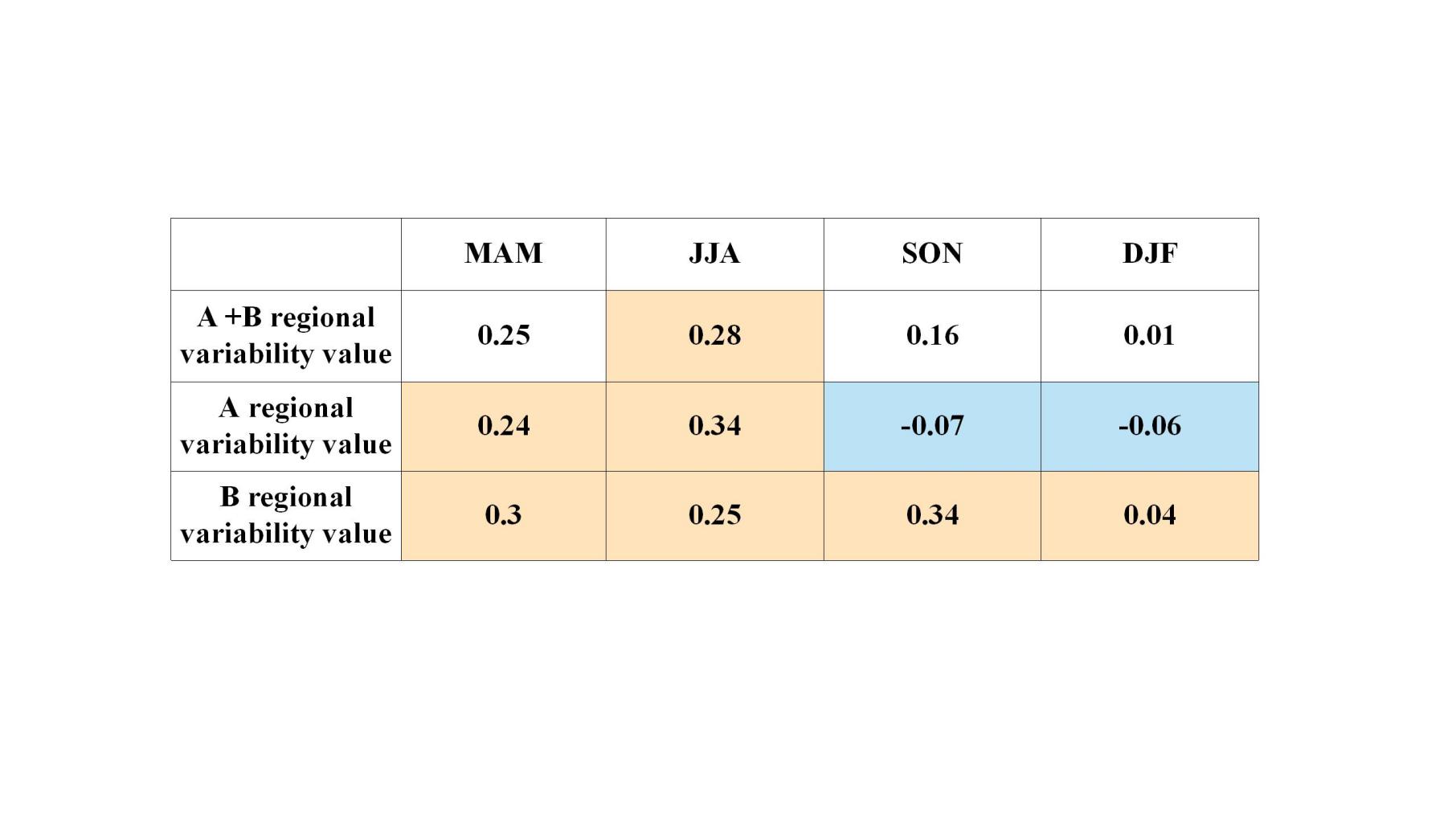


Table S1: Precipitation variabilities in area A, area B and area A + B in spring, summer, autumn and winter from 1991 to 2020 (mm/10 yr).

Figure S2


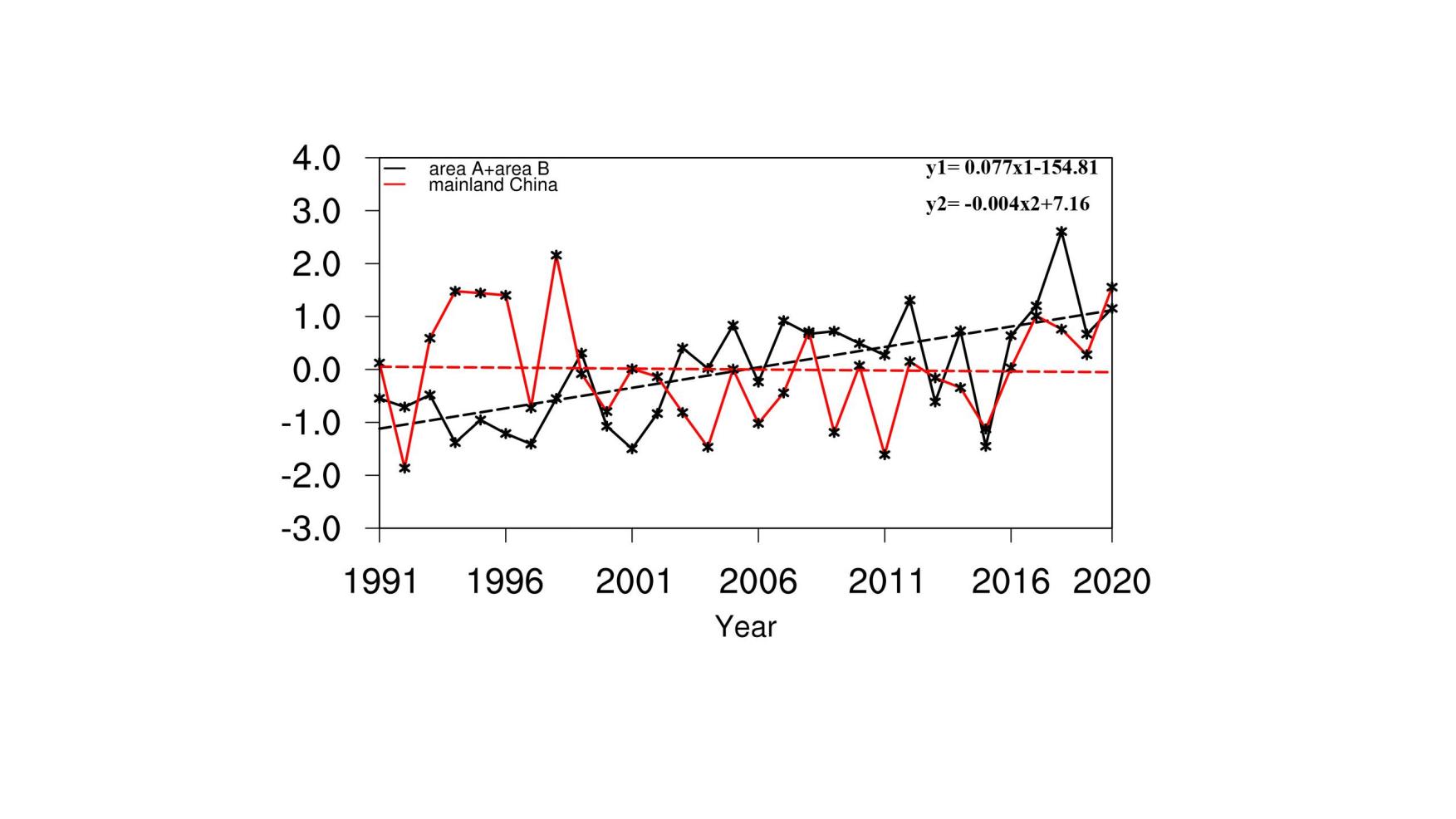


Figure S2: 1991–2020 summer interannual variation curves of standardized precipitation in A and B areas and mainland China.(solid line: interannual variation curve of precipitation; dotted line: trend line of precipitation ).

Figure S3


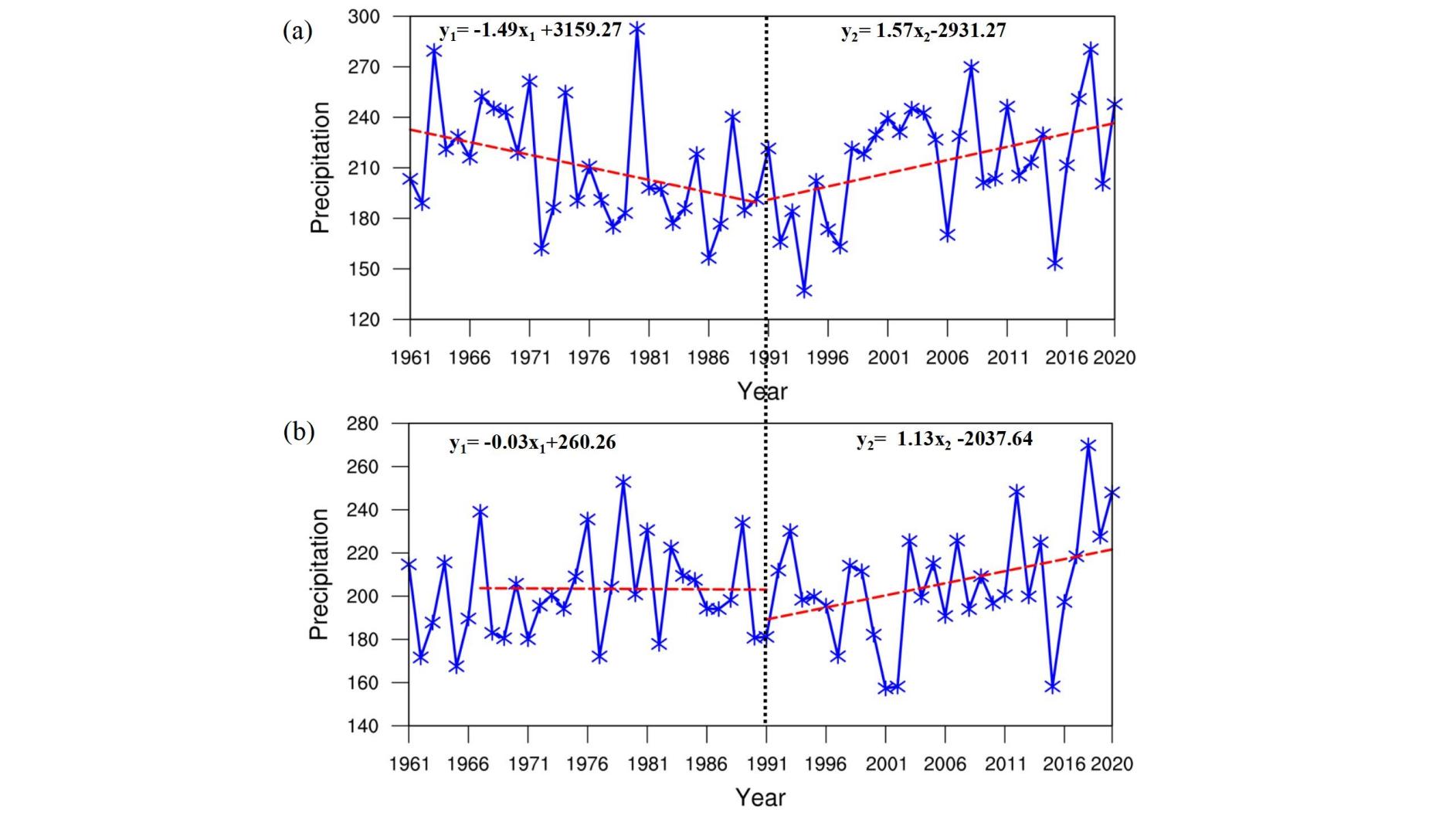


Figure S3: Interdecadal variation curves of summer precipitation in (a) area A and (b) area B from 1961 to 2020.

Figure S4


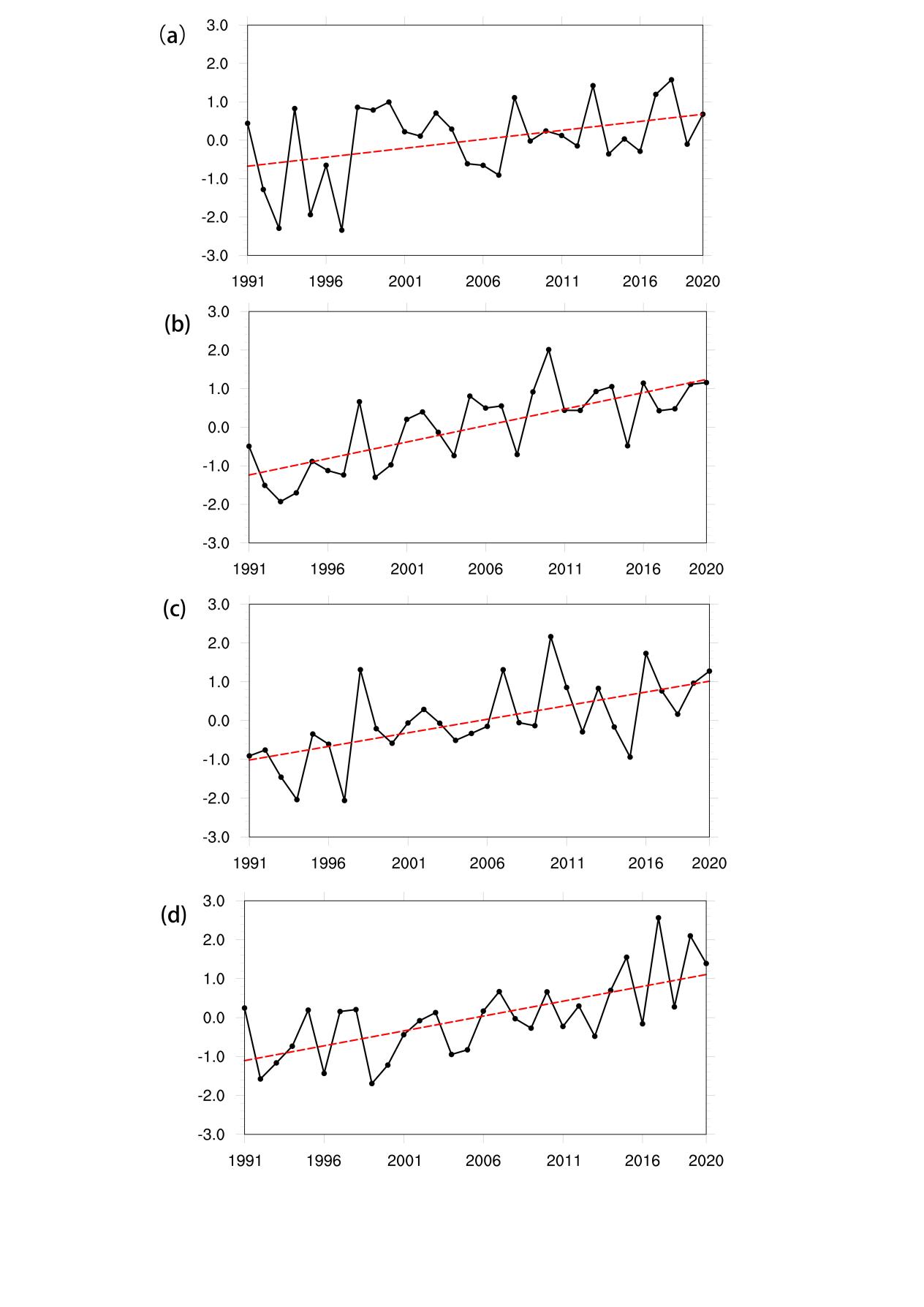


Figure S4: summer of 1991–2020: standardized interannual variation curves of specific humidity at 2 m in the SST high-impact areas (a) SST area 1, ( b) SST area 2, ( c) SST area 3, and (d) SST area 4.

Figure S5


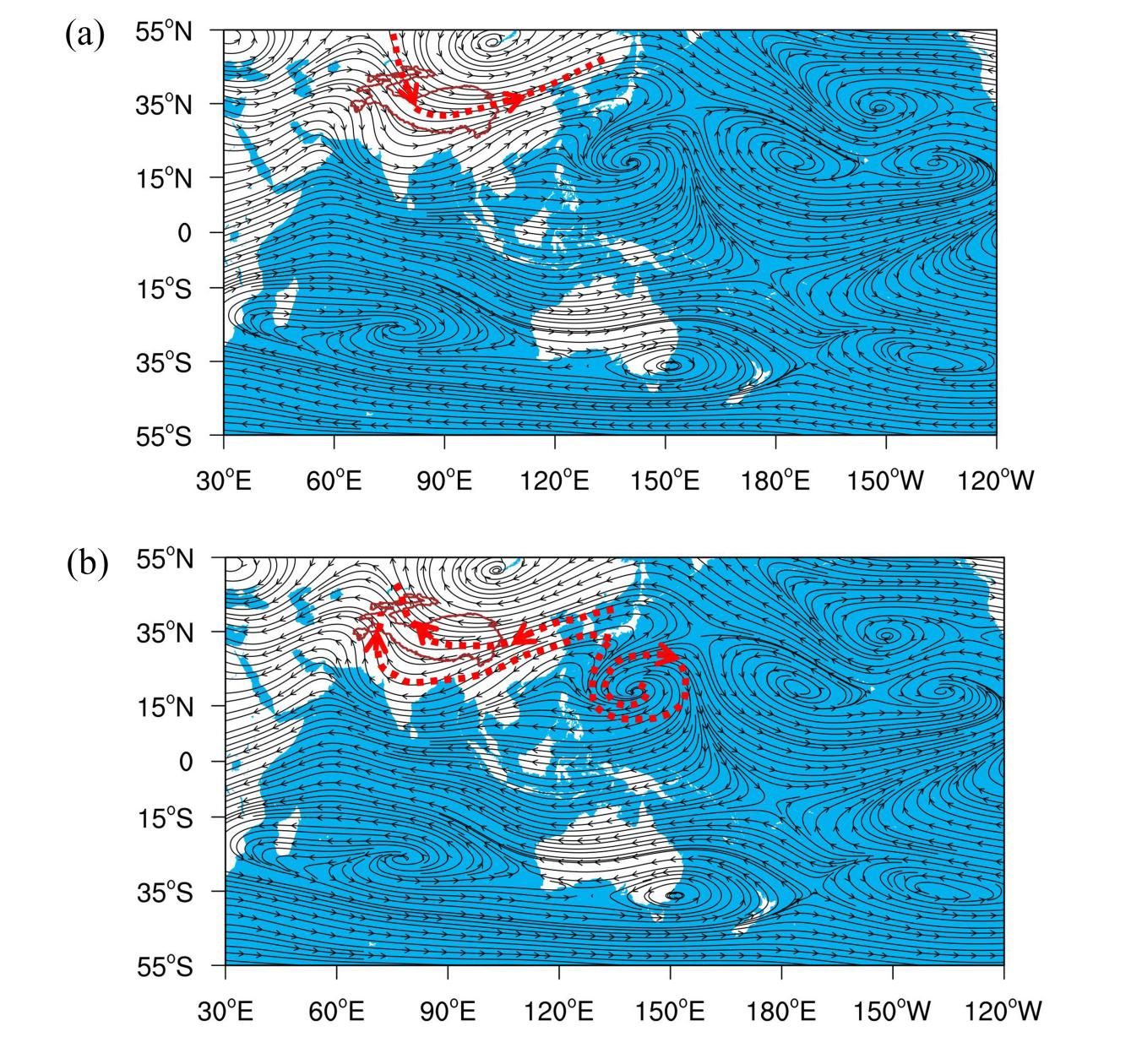


Figure S5: 500 hPa anomaly field of the whole-layer water vapor fluxes (a) for 1961-1990 and (b) for 1991-2020. The maps were generated with NCAR Command Language (NCL) Version 6.2.1 (http://www.ncl.ucar.edu/).
